# Validation of Visual and Auditory Digital Markers of Suicidality in Acutely Suicidal Psychiatric Inpatients: Proof-of-Concept Study

**DOI:** 10.2196/25199

**Published:** 2021-06-03

**Authors:** Isaac Galatzer-Levy, Anzar Abbas, Anja Ries, Stephanie Homan, Laura Sels, Vidya Koesmahargyo, Vijay Yadav, Michael Colla, Hanne Scheerer, Stefan Vetter, Erich Seifritz, Urte Scholz, Birgit Kleim

**Affiliations:** 1 Research and Development AiCure New York, NY United States; 2 Psychiatry New York University School of Medicine New York, NY United States; 3 Department of Psychology University of Zurich Zurich Switzerland; 4 Department of Psychiatry, Psychotherapy and Psychosomatics University of Zurich Zurich Switzerland; 5 Neuroscience Centre Zurich University of Zurich Zurich Switzerland

**Keywords:** digital phenotyping, digital biomarkers, digital health, depression, suicidal ideation, digital markers, digital, facial, suicide, suicide risk, visual, auditory

## Abstract

**Background:**

Multiple symptoms of suicide risk have been assessed based on visual and auditory information, including flattened affect, reduced movement, and slowed speech. Objective quantification of such symptomatology from novel data sources can increase the sensitivity, scalability, and timeliness of suicide risk assessment.

**Objective:**

We aimed to examine measurements extracted from video interviews using open-source deep learning algorithms to quantify facial, vocal, and movement behaviors in relation to suicide risk severity in recently admitted patients following a suicide attempt.

**Methods:**

We utilized video to quantify facial, vocal, and movement markers associated with mood, emotion, and motor functioning from a structured clinical conversation in 20 patients admitted to a psychiatric hospital following a suicide risk attempt. Measures were calculated using open-source deep learning algorithms for processing facial expressivity, head movement, and vocal characteristics. Derived digital measures of flattened affect, reduced movement, and slowed speech were compared to suicide risk with the Beck Scale for Suicide Ideation controlling for age and sex, using multiple linear regression.

**Results:**

Suicide severity was associated with multiple visual and auditory markers, including *speech prevalence* (*β*=−0.68, *P*=.02, *r*^2^=0.40), *overall expressivity* (*β*=−0.46, *P*=.10, *r*^2^=0.27), and head movement measured as *head pitch variability* (*β*=−1.24, *P*=.006, *r*^2^=0.48) and *head yaw variability* (*β*=−0.54, *P*=.06, *r*^2^=0.32).

**Conclusions:**

Digital measurements of facial affect, movement, and speech prevalence demonstrated strong effect sizes and linear associations with the severity of suicidal ideation.

## Introduction

Timely, efficient, sensitive, and noninvasive measurement approaches are required to improve suicide risk assessment [1]. One promising avenue is the use of remote digital monitoring methodologies that utilize smart devices, such as phones and wearables, in conjunction with deep learning algorithms to infer behavioral and physiological states associated with suicide risk [2,3].

While suicide risk is often comorbid with other mental disorders, in particular, major depressive disorder (MDD) and schizophrenia, suicidal behavior is increasingly recognized as unique in presentation and risk profile [4-7]. Based on prior knowledge, visual and auditory data sources represent a compelling direction in the objective measurement of behavior associated with suicide risk. Emil Kraepelin first observed that suicide risk was associated with melancholic states characterized by slowed speech, where patients appeared to “become mute in the middle of a sentence,” and further observed how “the facial expression and the general attitude are sleepy and languid, the speech is low…” [8]. More contemporarily, reduced facial expressivity and movement measured using standardized coding schemes based on videos of patient interviews differentiated depressed patients with and without suicide risk [9], and altered vocal characteristics have been observed in acutely suicidal patients [5].

A number of visual and auditory characteristics can be directly quantified, including gross motor activity [10], head movement variability [11-13], facial activity [14], and properties of speech [15]. The automated measurement of these clinical features introduces the possibility of objective digital assessment of visual and auditory markers of suicide risk. Given that audio and video data sources can be captured remotely, this further introduces the possibility of greatly scaling the reach and frequency of assessment. Increased scale and objectivity can facilitate increased accuracy and accessibility of clinical risk and treatment response assessment.

Visual and auditory biomarkers were selected based on a mechanistic theory that reduced serotonin, a key risk factor for suicidal behavior and a primary biological target for treatment of MDD, will affect behavioral characteristics, including an individual’s speech, head movement, and facial activity. Serotonergic tone is known to mechanistically impact motor functioning directly and via interactions with dopamine and norepinephrine signaling [16-18]. Depleted serotonin has been observed in the postmortem brains of individuals with MDD [19] and those who have committed suicide [20]. Furthermore, direct pharmacological manipulation of serotonin using selective serotonin reuptake inhibitors (SSRIs) increases suicide risk [21,22].

While novel measurements are promising, validation is required before such metrics can be interpreted clinically. Key steps for validation include comparison to traditional clinical measures, both cross-sectionally and as they change alongside treatment and disease course [23]. Such measures should strive to be easy to collect, should have increased sensitivity to facilitate frequent and accurate assessment, and should be validated in relationship to narrower biological phenotypes and treatment targets than traditional endpoints offer. This will lead to improved dynamic treatment research and clinical decision making based on modulation of underlying neurobiological deficits [24,25].

In this study, we examined measurements extracted from video interviews using open-source deep learning algorithms to quantify facial, vocal, and movement behaviors in relation to suicide risk severity in patients interviewed soon after admission to a psychiatric hospital following a suicide attempt.

## Methods

### Study Participants

Participants were recruited from the Psychiatric University Hospital, Zurich, Switzerland, as part of the “SIMON–Suicide Ideation MONitoring” study. The SIMON study has been funded by the Digital Lives initiative of the Swiss National Science Foundation (SNSF) and was approved by the ethics committee of the Faculty of Philosophy, University of Zurich (approval number: 19.2.1). The study aims to develop a digital protocol to monitor and predict suicidal ideation and hospital readmission in high-risk psychiatric patients. Digital markers of psychopathology are set to be derived from smartphone-based experience sampling, mobile passive sensing, and video recordings of patient free speech.

Patients were included in the study if they met the following criteria: (1) admission to the hospital after a suicide attempt or in the context of suicidal ideation, and suicidal ideation was identified in the first diagnostic intake interview, (2) sufficient knowledge of the German language, (3) being a smartphone user, and (4) discharge from the clinic after identification of suicidal ideation, with established outpatient care contact with a physician or psychologist. Patients were excluded if they met the following criteria: (1) planning to leave the greater Zurich area within the study period, (2) sharing a smartphone with another person, and (3) being active military personnel. Researchers kept track of all patients admitted to the hospital and contacted the treating psychologist or physician in case of eligibility. Patients who met the inclusion criteria and for whom an approval from the psychologist or physician was granted were informed about the study. If patients agreed to participate in the study, they were invited for a baseline assessment appointment that entailed the following: (1) detailed information about the study, (2) collection of informed consent signed by the patient, (3) assessment of current mental disorders with the Mini International Neuropsychiatric Interview (MINI; version 6), (4) a short videotaped semistructured qualitative interview (for which additional informed consent signed by the patient was obtained) upon agreement, (5) electronic/pen and paper questionnaires evaluating relevant psychological variables, and (6) smartphone app installation. Participants were reimbursed with up to CHF 120 (US $127).

At the time of the video assessment, all patients had an inpatient status at the Psychiatric University Hospital Zurich. We recruited patients admitted to the psychiatric hospital to ensure an appropriate reach and a sufficient sample size of the high-risk psychiatric patient group. For practical reasons and because of the format of the semistructured interviews (ie, questions asked by the researcher and video recordings with a tablet), we obtained video recordings during the baseline interview, when patients were still inpatients. However, the remaining parts of the study (ie, remote patient monitoring using smartphones) commenced after patients were discharged from the hospital. In that way, we assessed patient well-being and behavior in an ecologically valid manner outside the hospital. However, this study only focused on the video recordings from the time patients were inpatients.

Participants were recruited for the study on a rolling basis. At the time of the analysis, 30 patients agreed to participate in the videotaped interview, of whom 20 completed the necessary baseline questionnaires. Ultimately, the analysis was conducted on a sample of 20 patients.

### Clinical Assessment of Suicidality

Assessment of suicide risk was completed at baseline. The Beck Scale for Suicide Ideation (BSS) questionnaire (German validated version) [26] was administered to evaluate patients’ current intensity of attitudes, plans, and behaviors to commit suicide. Patients’ history of nonsuicidal self-injury and suicide attempts was assessed using the following two self-report items from the Self-Injurious Thoughts and Behaviors Interview (SITBI): (1) “In your life, have you purposefully hurt yourself without wanting to die?” and (2) “How many times in your lifetime have you made an attempt to kill yourself during which you had at least some intent to die?” [27,28].

### Collection of Video and Audio Data

At baseline, patients were given the choice of additionally participating in a video-recorded interview. Upon agreement, patients signed an informed consent form specific to this part of the study. A short videotaped semistructured qualitative interview was performed. A laptop was placed on the table in front of the patient, and 1-minute video and audio samples were recorded. During the qualitative video interview, participants answered introductory questions assessing their current state, as well as questions about experiences with different valences (ie, neutral, positive, and negative) and temporal dimensions (ie, past, present, and future). Overall, the following six video and audio samples, each at 1 minute, were recorded per participant: introduction (neutral present), neutral, positive past, positive future, negative past, and negative future. Multimedia Appendix 1 displays exemplary questions asked for each category. The conversation for each category starts with the central question (first in order) asked by the researcher, followed by additional questions to keep the participant involved and talking during the 1-minute recordings.

### Machine Learning Computer Vision and Voice Analysis

All analyses were conducted in a Python environment using open-source tools. No novel machine learning models were trained for the purposes of this study, rather existing tools for the measurement of facial expressivity [29], vocal acoustics [30], and patterns of movement [31] were utilized. The code used to calculate the biomarkers has been compiled into its own open-source package, which allows for other researchers to replicate the methods used in this manuscript [32]. The videos were segmented to only include the participants’ responses to the questionnaires, with behaviors during free speech cropped out and then concatenated for analysis of digital markers.

#### Facial Activity Analysis

Initially, all videos were segmented into frames, resulting in a minimum of 33 image frames per second. Thereafter, OpenCV, an open-source computer vision software package [33], was used to segment each image into three matrices consisting of red, blue, and green spectrum pixels. Subsequently, each frame was analyzed using OpenFace [29], an open source software package that has demonstrated validity next to expert human ratings of Facial Action Coding Scheme (FACS) [34], a standardized methodology to measure facial movements that reflect activity in the underlying human facial musculature utilized in the production of basic emotions. Specifically, for each frame, OpenFace outputs (1) a binary value indicating the presence of an action unit and (2) a continuous value indicating the intensity with which the action unit is being expressed. Action unit intensities were used to derive measures of individual emotional expressivity (ie, *happiness expressivity*, *sadness expressivity*, *fear expressivity*, *anger expressivity*, *surprise expressivity*, and *disgust expressivity*). Action unit intensities were also used to calculate *overall expressivity* regardless of emotion.

#### Movement Analysis

The angle of the head’s pitch (up and down movement) and yaw (side to side movement) was acquired for each video frame using OpenFace. The standard deviations of the frame-wise pitch and yaw measurements were used as indicators of head movement (ie, *head pitch variability* and *head yaw variability*).

#### Voice Analysis

Recordings were segmented into speech and nonspeech parts using Parselmouth, an open-source software package utilized for vocal analysis [30]. The percentage of the audio file where speech was recorded as opposed to no speech (ie, *speech prevalence*) was calculated to represent how much the participant was talking given the length of the recording.

### Data Analysis

Initially, BSS scores were regressed on all movement, facial, and audio markers controlling for age and sex using separate multiple linear regressions. Facial activity, movement, and voice are all behavioral characteristics that are influenced by both age and sex. Hence, it was important to conduct a multiple linear regression controlling for age and sex in order to remove the influence of those factors on the final comparisons between digital measures and BSS scores. In addition to significance testing, unique variance accounted for in BSS scores by the digital measures was assessed using Cohen *d* [35]. Following analysis of *overall expressivity*, the BSS was regressed on expressivity of each emotion correcting for multiple comparisons using false discovery rate adjustment to reduce *P*-value inflation due to chance.

## Results

### Suicide Severity

Suicidal ideation severity on the BSS across subjects was, on average, above the clinical cutoff of 9, indicating severe suicide risk (*µ*=10.9, σ=10.2, range 0-31) and an average count of three past suicide attempts (σ=6.9, range 0-30).

### Facial Activity

Controlling for age and sex, *overall expressivity* demonstrated a significant negative linear association with BSS scores, indicating that decreased facial activity is associated with greater suicide risk (*β*=−0.46, *P*=.01, *r*^2^=0.27). This indicates that facial activity decreases as suicide severity increases, with the strongest evidence in the context of facial expressions without emotional expressions.

To better understand if particular emotions contributed to the observation of *composite expressivity*, we regressed BSS scores with individual emotional expressivity, sex, and age. *P* values were adjusted using false discovery rate adjustment. Post-hoc analyses demonstrated significance using Benjamini-Hochberg adjusted *P* values for *sadness expressivity* (*β*=−0.68, *P*=.01, *r*^2^=.43), *surprise expressivity* (*β*=−0.74, *P=*.002, *r*^2^=0.53)*,* and *disgust expressivity* (*β*=−0.64, *P=*.04, *r*^2^=0.35), but not for *fear*, *anger*, and *happiness expressivity* (Table 1).

**Table 1:** Results from multiple linear regression between Beck Scale for Suicide Ideation questionnaire scores and digital measurements of facial expressivity, vocal behavior, and head movement, controlling for age and sex.

Predictor			Coefficient		Standard error		*t* (df)		*P* value		*F* (df)		R^2^		Adjusted R^2^		*P* value
**Regression 1**											0.9013 (1)		0.153		−0.017		.46
	Constant	0.2020		0.380		0.531 (18)		.60									
	Anger expressivity	−0.2456		0.322		−0.762 (18)		.46									
	Age	0.0068		0.007		0.960 (18)		.35									
	Sex	0.1167		0.172		0.680 (18)		.51									
**Regression 2**											2.6930 (1)		0.350		0.220		.08
	Constant	0.1213		0.240		0.506 (18)		.62									
	Disgust expressivity	−0.6401		0.278		−2.305 (18)		.04									
	Age	0.0120		0.006		1.980 (18)		.07									
	Sex	0.1422		0.146		0.972 (18)		.35									
**Regression 3**											1.0430 (1)		0.173		0.007		.40
	Constant	0.2628		0.380		0.692 (18)		.50									
	Fear expressivity	−0.3210		0.328		−0.978 (18)		.34									
	Age	0.0060		0.007		0.842 (18)		.41									
	Sex	0.1056		0.170		0.620 (18)		.54									
**Regression 4**											0.6834 (1)		0.120		−0.056		.58
	Constant	0.0051		0.292		0.018 (18)		.99									
	Happiness expressivity	−0.0228		0.255		−0.089 (18)		.93									
	Age	0.0083		0.007		1.194 (18)		.25									
	Sex	0.1517		0.178		0.852 (18)		.41									
**Regression 5**											3.7140 (1)		0.426		0.311		.04
	Constant	0.4394		0.270		1.629 (18)		.12									
	Sadness expressivity	−0.6785		0.240		−2.830 (18)		.01									
	Age	0.0082		0.006		1.480 (18)		.16									
	Sex	0.0345		0.152		−0.228 (18)		.82									
**Regression 6**											5.7130 (1)		0.533		0.440		.008
	Constant	−0.0441		0.198		−0.223 (18)		.83									
	Surprise expressivity	−0.7437		0.204		−3.645 (18)		.002									
	Age	0.0143		0.005		2.734 (18)		.02									
	Sex	0.2421		0.127		1.912 (18)		.08									
**Regression 7**											1.8200 (1)		0.267		0.120		.19
	Constant	0.2881		0.300		0.961 (18)		.35									
	Overall expressivity	−0.4585		0.264		−1.734 (18)		.10									
	Age	0.0054		0.006		0.833 (18)		.42									
	Sex	0.1947		0.158		1.234 (18)		.24									
**Regression 8**											3.3890 (1)		0.404		0.285		.046
	Constant	0.3291		0.256		1.285 (18)		.22									
	Speech prevalence	−0.6808		0.255		−2.674 (18)		.02									
	Age	0.0057		0.006		0.991 (18)		.34									
	Sex	0.3429		0.158		2.170 (18)		.047									
**Regression 9**											4.5330 (1)		0.476		0.371		.02
	Constant	0.4192		0.248		1.689 (18)		.11									
	Head pitch variability	−1.2465		0.391		−3.189 (18)		.006									
	Age	0.0124		0.005		2.294 (18)		.04									
	Sex	−0.0342		0.143		−0.239 (18)		.81									
**Regression 10**											0.1170 (1)		0.317		0.181		.12
	Constant	0.1006		0.245		0.411 (18)		.69									
	Head yaw variability	−0.5408		0.260		−2.081 (18)		.06									
	Age	0.0125		0.006		1.979 (18)		.07									
	Sex	0.1723		0.150		1.146 (18)		.27									

### Voice Analysis

Controlling for age and sex, *speech prevalence* demonstrated a significant negative linear association with BSS scores, indicating that suicide severity scores increased as speech decreased (*β*=−0.68; *P*=.02, *r*^2^=0.40; Table 1).

### Movement Analysis

Controlling for age and sex, both *head pitch variability* and *head yaw variability* demonstrated significant negative linear relationships with suicide severity (*β_pitch_*=−1.24, *P*=.006, *r*^2^=0.48; *β_yaw_*=−0.54, *P*=.055, *r*^2^=0.32), indicating that lower levels of head movement were associated with greater suicide severity scores (Table 1).

## Discussion

We examined visual and auditory measures of facial activity, head movement, and speech production, which were all calculated using deep learning algorithms applied to open-ended clinical interviews with psychiatric patients following a suicide attempt. The goal of this work was to determine if key indicators of suicide severity could be measured in an objective and automated manner using video data captured during clinical interviews that provided structured questions, but were otherwise kept deliberately open to mimic psychiatric interviewing in routine care. Achieving this goal provides a proof of concept that suicide risk can be assessed through analysis of unstructured video interview data in conjunction with deep learning algorithms designed to measure clinical/behavioral characteristics.

Objective measurements of visual and auditory markers of suicidality calculated from videos of patient behavior can be useful in the context of psychiatric care and clinical research, in particular, if the measurements can be acquired without specialized hardware using video or audio from regular patient-clinician interactions, as has been demonstrated in this study. Such tools have applications in clinical practice as they can allow for remote measurement of symptomatology. For example, virtual clinic visits through video calls can involve digital measurements to assess mental health if such measurements are integrated into the technology utilized. Existing smartphone-based platforms allow for the collection of video and audio data and subsequent calculation of digital measures, and can be applied in out-patient settings to remotely acquire these measurements without adding further burden on the clinical staff [36-38]. Of course, such technologies face a great deal of challenges before they can be deployed in patient care, starting with further validation of methodologies, passage through regulatory review as dependable clinical measures, and adoption by health care professionals into their day-to-day clinical workflows [39]. Ultimately, methods that can provide remote, objective, and passive measurement of clinical functioning can greatly increase the reach of treatment development, dissemination, and personalization [25,40].

Finally, the studied markers were selected based on a mechanistic theory that reduced serotonin in the brain, a key risk factor for suicidal behavior, will manifest in behavioral characteristics, including those involving a patient’s speech, head movement, and facial activity. These primary hypotheses were confirmed. Overall facial expressivity, speech activity, and head movement during spontaneous behavior, which are all downstream effects of motor slowing, were correlated with BSS scores. This work provides a proof of concept that proxy markers of underlying neurobiological functioning can be used to measure clinical risk. Demonstrating this indicates that there is potential to remotely measure more specific neurobiological phenotypes to determine risk or examine the response to treatment. For example, owing to suicide risk associated with SSRIs, there is a need for close clinical monitoring during the initiation of treatment. Similarly, such models may be useful to determine who would benefit from treatments that affect serotonergic tone. Ultimately, by measuring a more specific biologically based phenotype, there is greatly increased opportunity to improve sensitivity of measurement and specificity of treatment [24].

The work has key limitations. First, this work represents a proof of concept. The indicated markers for risk assessment or measurement of clinical severity should only be used in an exploratory manner until such markers can be validated in larger and more diverse clinical populations and settings. We need to directly determine the amount of video data needed and the clinically meaningful cutoffs to determine clinical functioning. It is likely that like traditional suicide risk assessment, multiple characteristics together are needed to determine risk or severity. Multiple digital measures can be combined to produce a more robust metric, but like traditional scale development, this requires larger samples from diverse populations. Importantly, the current work uses open-source software, and additionally, we have published our software methods. As such, there is no access barrier for researchers to implement identical methods to test and extend the current approach. An open-source approach lends itself to rapid replication, extension, and implementation.

Ultimately, this proof-of-concept study demonstrates that theoretically grounded visual and auditory digital measurements are valid as markers of suicide severity. This effort provided a digital approach akin to traditional clinical assessment whereby a skilled clinician listens to a patient and applies internal working models developed through years of experience to assess clinical functioning.

## Appendix

Multimedia Appendix 1 Exemplary questions for the six categories of the videotaped semistructured qualitative interview.

## Abbreviations

BSS: Beck Scale for Suicide Ideation
MDD: major depressive disorder
SSRI: selective serotonin reuptake inhibitor
