# Supplementary material for: Validation of Visual and Auditory Digital Markers of Suicidality in Acutely Suicidal Psychiatric Inpatients: Proof-of-Concept Study
Source: J Med Internet Res. 2021 Jun 3;23(6):e25199. doi: 10.2196/25199 (PMC8212625; doi:10.2196/25199)
Supplement: Multimedia Appendix 1 [file jmir_v23i6e25199_app1.docx]

**Multimedia Appendix 1.** Exemplary questions for the six categories of the videotaped semistructured qualitative interview.

| Category | Exemplary questions |
| --- | --- |
| Introduction | - How are you?  - How's your day so far?  - Can you please tell me about your day, starting from when you woke up? |
| Neutral | - Can you remember a TV show or a movie you saw recently?  - What was the name of the show/movie?  - When did you watch it?  - Why did you choose it?  - What happened in the show/movie? Please describe the story as best you can.  - Who were the most important persons/lead actors?  - Can you tell me what the motivation of these people was?  - How did the show/movie end? |
| Positive past | - Can you remember an event that made you really happy?  - Who else but you was involved?  - Can you tell me what led to that event?  - Can you tell me as best you can what happened, as if it was a story?  - Can you tell me how it ended?  - Are you still happy when you think about that event? If so, why or why not? |
| Positive future | - Is there an event in the coming days that you are looking forward to?  - Can you tell me about that event?  - Why are you looking forward to it?  - Who else but you is involved?  - Can you tell me what leads to this event? |
| Negative past | - Can you remember an event that was disturbing or stressful for you? (*participants are informed that this question does not relate to traumatic events they might have experienced).  - Who else but you were involved?  - Can you tell me what led to this event?  - Can you tell me as best you can what happened, as if it were a story?  - Can you tell me how it ended?  - Are you feeling better about that now, or is it still upsetting for you? If so, why or why not? |
| Negative future | - Is there an event in the coming days that you are not looking forward to or even are afraid of?  - Can you tell me about this event?  - Why are you not looking forward to it?  - Who else but you is involved?  - Can you tell me what leads to this event? |
